# Quantification of nisin concentration from fluorescence‐based antimicrobial activity assay using Bayesian calibration

**DOI:** 10.1002/btpr.3495

**Published:** 2024-07-26

**Authors:** Valentin Steier, Michael Osthege, Laura M. Helleckes, Maximilian Siska, Eric von Lieres, Wolfgang Wiechert, Sebastian J. Reich, Christian U. Riedel, Marco Oldiges

**Affiliations:** ^1^ Institute of Bio‐ and Geosciences, IBG‐1: Biotechnology Forschungszentrum Jülich GmbH Jülich Germany; ^2^ Institute of Biotechnology RWTH Aachen University Aachen Germany; ^3^ Computational Systems Biotechnology (AVT.CSB) RWTH Aachen University Aachen Germany; ^4^ Department of Biology Ulm University Ulm Germany

## Abstract

Bacteriocins are ribosomally synthesized peptides with the innate ability to kill or inhibit growth of other bacteria. In recent years, bacteriocins have received increased interest, as their antimicrobial activity enhances food safety and shelf life by combatting pathogens such as *Listeria monocytogenes*. They also have application potential as an active pharmaceutical compound to combat multidrug‐resistant pathogens. As new bacteriocins continue to be discovered, accelerated workflows for screening, identification, and process development have been developed. However, antimicrobial activity measurement is often still limited with regards to quantification and throughput. Here, we present the use of a non‐linear calibration model to infer nisin concentrations in cultivation supernatants of *Lactococcus lactis* ssp. *lactis* B1629 using readouts of pHluorin2 fluorescence‐based antimicrobial activity assays.

## 1 INTRODUCTION

Bacteriocins are ribosomally synthesized peptides with narrow or broad antimicrobial activity spectra against bacteria.^1^ They have long been used to prevent contamination with pathogens in food products and have recently garnered increased interest due to their capability to combat pathogens exhibiting antimicrobial resistance or biofilm formation.^2^ The most prominent bacteriocin is nisin, having been discovered in 1933.^3^ It is produced by several *Lactococcus lactis* and *Streptococcus* strains^4^ and is widely used in food products and for treatment of veterinary diseases.^5^ The identification and characterization of novel bacteriocins from microbial sources requires the confirmation of antimicrobial activity against a certain susceptible indicator strain. The academic standard are agar plate‐based inhibition assays,^6^ which can be easily carried out, require little equipment and are inexpensive, but often lack reproducibility and reliability with regards to quantification.^7^ Optical measurement alternatives enhancing the quantitative properties were developed, such as the pHluorin2 assay that uses ratiometric fluorescence readouts to quantify antimicrobial activity via induced pore formation.^8^ Despite the enhanced reliability of the pHluorin2 assay, some general drawbacks of antimicrobial activity measurements remain. Quantification of antimicrobial activity is often performed via threshold‐based categorization, such as minimal inhibitory dilution or minimal inhibitory concentration.^9^, ^10^, ^11^ Bacteriocin standards are only available in few selected cases, leading to either costly chemical synthesis or standard‐independent categorization. Even with available standards, calibrations are often simplified and rarely reflect the usually non‐linear mathematical relationship between bacteriocin concentration and resulting antimicrobial activity.^12^, ^13^ Moreover, due to their laborious nature, data from agar plate‐based inhibition assays often lack replicates, hindering error estimation or uncertainty quantification.

Bayesian calibration models have been employed in biotechnology as a more accurate way to quantify independent variables (e.g., concentrations) based on measurement values (dependent variable), and have the inherent property of uncertainty quantification based on deviation of readouts from the calibration function.^14^ Common use cases may be biomass calibration based on optical measurements,^15^ quantification of enzyme activities^16^ or metabolites such as glucose or amino acids.^14^ In this work, we set up a non‐linear calibration model to quantify nisin concentrations in cultivation supernatants of *Lactococcus lactis* ssp. *lactis* based on readouts from the fluorescence‐based pHluorin2 assay and demonstrate the applicability of the calibration model. Moreover, we highlight the advantages of using non‐linear calibration compared to a standard threshold‐based quantification approach.

## 2 METHODS

### 2.1 Strains and cultivation parameters

Nisin Z‐producing *Lactococcus lactis* ssp. *lactis* B1629^17^ was cultivated in M17 complex medium^18^ (Merck). For cryo‐conservation, the strain was grown from a single colony, incubated statically at 30°C, resuspended in 0.9% (w/v) NaCl solution with 25% glycerol at an optical density (OD) of 10 at 600 nm and stored at −80°C. Pre‐cultures were carried out in 50 mL centrifugation tubes with 30 mL M17 medium (0.5% glucose), inoculated to an OD of 0.1 and incubated statically at 30°C for 16 h. Pre‐cultures were centrifuged (4000g, 4°C, 10 min) and resuspended in 0.9% (w/v) NaCl solution prior to main culture inoculation to an OD of 0.1. Main cultures were cultivated in a parallelized microbioreactor (BioLector Pro, Beckman Coulter) at 30°C, 600 rpm, and 3 mm shaking diameter without humidity control. The used microtiter plate (MTP‐R48‐BOH 1, Beckman Coulter) was sealed with an adhesive non‐gas‐permeable aluminum foil. Integrated scattered light measurement was used to monitor growth.

Cultivations were carried out with different media compositions by supplementing different concentrations of glucose, yeast extract and MES buffer (pH 6.5) (Table 1). Cultivation samples were drawn manually and centrifuged at 21,500 g at 4°C for 10 min.

**TABLE 1:** Concentration of supplemented media components to M17 complex medium for cultivation of *L. lactis* B1629.

Condition/Sample	Glucose conc./g L^−1^	Yeast extract conc./g L^−1^	MES buffer conc./M	Mean max. backscatter/‐
S1	0	0	0	12
S2	10	0	0.2	52
S3	20	0	0.2	94
S4	10	10	0	70
S5	20	10	0	67
S6	5	20	0.2	57
S7	10	20	0.2	86
S8	20	20	0	70

### 2.2 pHluorin2 antimicrobial activity assay

The pHluorin2 assay was carried out largely as previously published^8^ using *Listeria innocua* LMG2785/pNZ‐pHin2^*Lm*^.^19^ Optimized listeria minimal buffer (LMBO) was used, containing 200 mM MES, 4.82 mM KH_2_PO_4_, 11.55 mM Na_2_HPO_4_, 1.7 mM MgSO_4_, 4.54 mM (NH_4_)_2_SO_4_, 55 mM glucose, and adjusted to a pH value of 6.2. All measurements were carried out using 100 μL of sensor strain cell suspension with an OD set to 3.0 and 100 μL sample. Cetrimonium bromide (CTAB) was used as positive control, LMBO was used as negative control and commercial nisin (Sigma‐Aldrich) was used as standard (0.01–5.0 μg mL^−1^). Emission at 520 nm was measured at excitation wavelengths of 400 and 480 nm. The calculated emission ratios of 400 and 480 nm excitation were divided by the mean emission ratio of the negative control (LMBO). Antimicrobial activity measurements of cultivation supernatants were carried out in serial two‐fold dilutions up to a dilution factor of 256 (2^8^). The calculations were carried out using Python, with the exact dependency versions listed in the accompanying code repository. Necessary dilutions of commercial nisin were carried out using a Tecan Freedom EVO 200 liquid handler and pipetting worklists were created using the robotools Python package.^20^

### 2.3 Calibration model

The calibration model was set up using the Python package calibr8 ^21^ using the generalized logistic model function. Fitting of the model parameters was performed using fluorescence data from pHlourin2 assay with 96 different concentrations of commercial nisin standard, covering a concentration range from 0.01–5.0 μg mL^−1^. Data, calibration model and demonstration workflow are provided as repository (https://github.com/JuBiotech/Supplement-to-Steier-et-al.-2024).

## 3 RESULTS

Quantification of bacteriocins is mainly hampered due to absence of commercial standards, with the exception of nisin. Recently, the metric of the bacteriocin unit (BU) was introduced to quantify the antimicrobial activity based on readouts from the pHluorin2 assay, which does not require pure bacteriocin standards.^22^ The pHluorin2 assay is a fluorimetric antimicrobial activity assay that measures the change in fluorescence ratio following membrane damage caused by bacteriocins. The membrane damage leads to a change in intracellular pH value, yielding changes in the fluorescence spectrum. Here, a low fluorescence ratio indicates a dead cell population, while a high fluorescence ratio indicates a live cell population.

**FIGURE 1:**
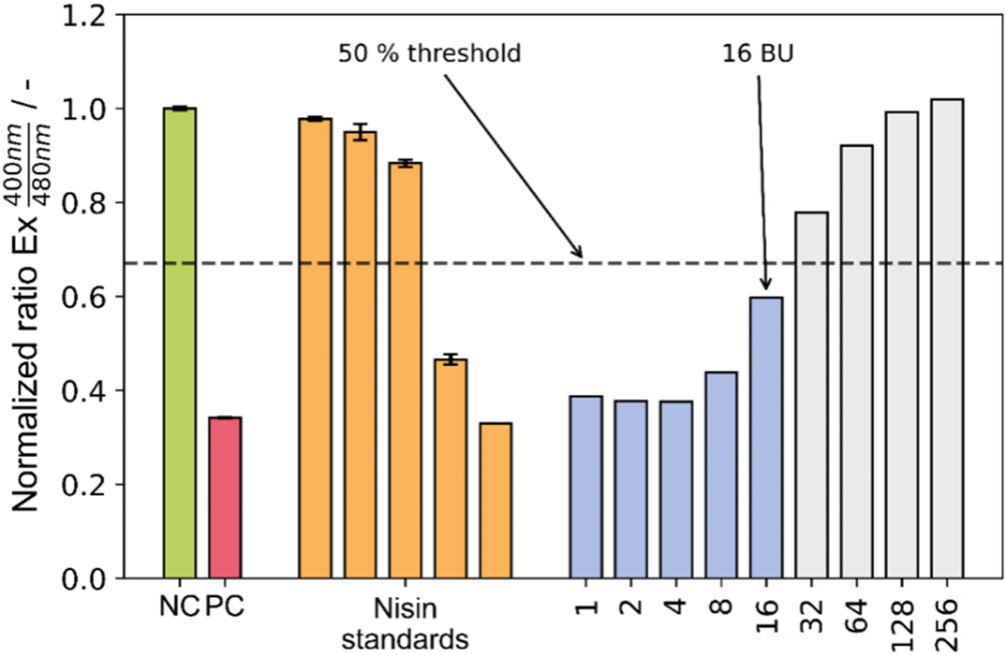
Exemplary readouts from pHluorin2 assay using sensor strain *L. innocua* LMG2785/pNZ‐pHin2^*Lm*^. NC: Negative control (LMBO buffer); PC: Positive control (LMBO containing 0.01% CTAB). Nisin standards: LMBO containing nisin in concentrations 0.128, 0.5, 1.25, 2.5, and 5 μg mL^−1^. 1–256 indicate dilution factors of cultivation supernatant from *L. lactis* B1629.

The BU method evaluates the necessary dilution to elicit an antimicrobial activity equivalent to a cell death signal of at least 50% of the cell population (see Figure 1). Instead of analyzing a dilution series for every tested sample, it provides a single BU value for each sample and allows quick and convenient comparison of large sample cohorts of different bacteriocin producers. The availability of commercial nisin standards enables not just BU analysis but also precise quantification through calibration, as demonstrated in other types of antimicrobial activity assays.^23^, ^24^ Here, the functional relationship between nisin concentration and its antimicrobial effect, given as measured fluorescence ratio, is clearly non‐linear (Figure 1).

In literature, the relationship between bacteriocin concentration and elicited antimicrobial activity has been reported to be of sigmoidal nature.^13^ Hence, linear or exponential/logarithmic regression are no suitable options for model fitting and quantification; although they are performed occasionally in literature.^25^, ^26^ Instead, we aimed for a logistic calibration model using the Python package calibr8, which facilitates Bayesian inference of the quantity of interest, in our case antimicrobial activity. The principle of such a calibration involves mathematically describing the trend of the relationship between a dependent variable (fluorescence ratio) and an independent variable (nisin concentration).^14^ A second mathematical function of constant, linear or other nature is used to describe the behavior of the measurement noise over the range of the independent variable.

**FIGURE 2:**
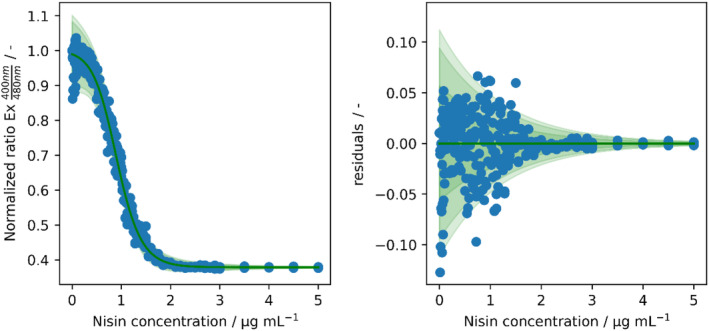
Non‐linear, heteroscedastic calibration model fitted to calibration data of nisin standard concentration (0.01–5 μg mL^−1^) and fluorescence readouts from pHluorin2 assay using sensor strain *L. innocua* LMG2785/pNZ‐pHin2^*Lm*^. Left: Generalized logistic calibration function fitted to measurements from 96 different nisin concentrations in triplicates. Right: Absolute residuals with respect to the modeled mean function. Green bands mark the 95%, 90%, and 68% likelihood bands of predicted ratios.

Here, we measured a total of 96 different concentrations between 0.01 and 5.0 μg mL^−1^ of commercial nisin in the pHluorin2 assay in triplicates and fitted a model using calibr8 (Figure 2). We chose a generalized logistic function to describe the relationship between nisin concentration and measured fluorescence ratio, since it covers the sigmoidal nature and also contains additional parameters for a more accurate description of the function (Figure 2, left). We chose an exponential function to describe the heteroscedastic relationship between nisin concentration and measurement noise, i.e. the observation that measurement errors are not constant with higher error at lower concentration (Figure 2, right).

The obtained fluorescence ratio from the dilution series yielded a clear sigmoidal curve (Figure 2, left) with normalized fluorescence ratios of about 1.0 for lowest nisin concentrations followed by a sigmoidal decrease to a plateau at a normalized ratio of about 0.4 for >2.5 μg mL^−1^ of nisin. Please note that a decrease in fluorescence ratio corresponds to an increase in dead cells. The residual plot (Figure 2, right) shows the deviation between the model prediction and observed measurement values. The green bands visualize the predicted spread of observations, and explain the distribution of measurement values well across the entire range of interest. The spread was small across most of the measured concentration range, observations are randomly distributed on both sides of the calibration function (Figure 2, right, green line), and were not systematically biased. This indicates that the generalized logistic function, combined with the exponential noise function, is an adequate model of the pHluorin2 assay, also in its saturations at <0.5 or >1.5 μg mL^−1^. Both functions are thoroughly documented in the calibr8 documentation (https://calibr8.readthedocs.io/en/latest/index.html) as well as the publication^14^ and code repository.^27^

Since the calibration shows that the pHluorin2 assay is only sensitive in a range of approximately 0.5–1.5 μg mL^−1^, the samples of culture supernatants with higher activities must be measured in appropriate dilution. To overcome the limitations of the threshold‐based approaches, it is necessary to include information from multiple dilutions. We implemented this by constructing a likelihood function that accounts for the different dilution factors of multiple samples. For details on the implementation see the accompanying code repository.

**FIGURE 3:**
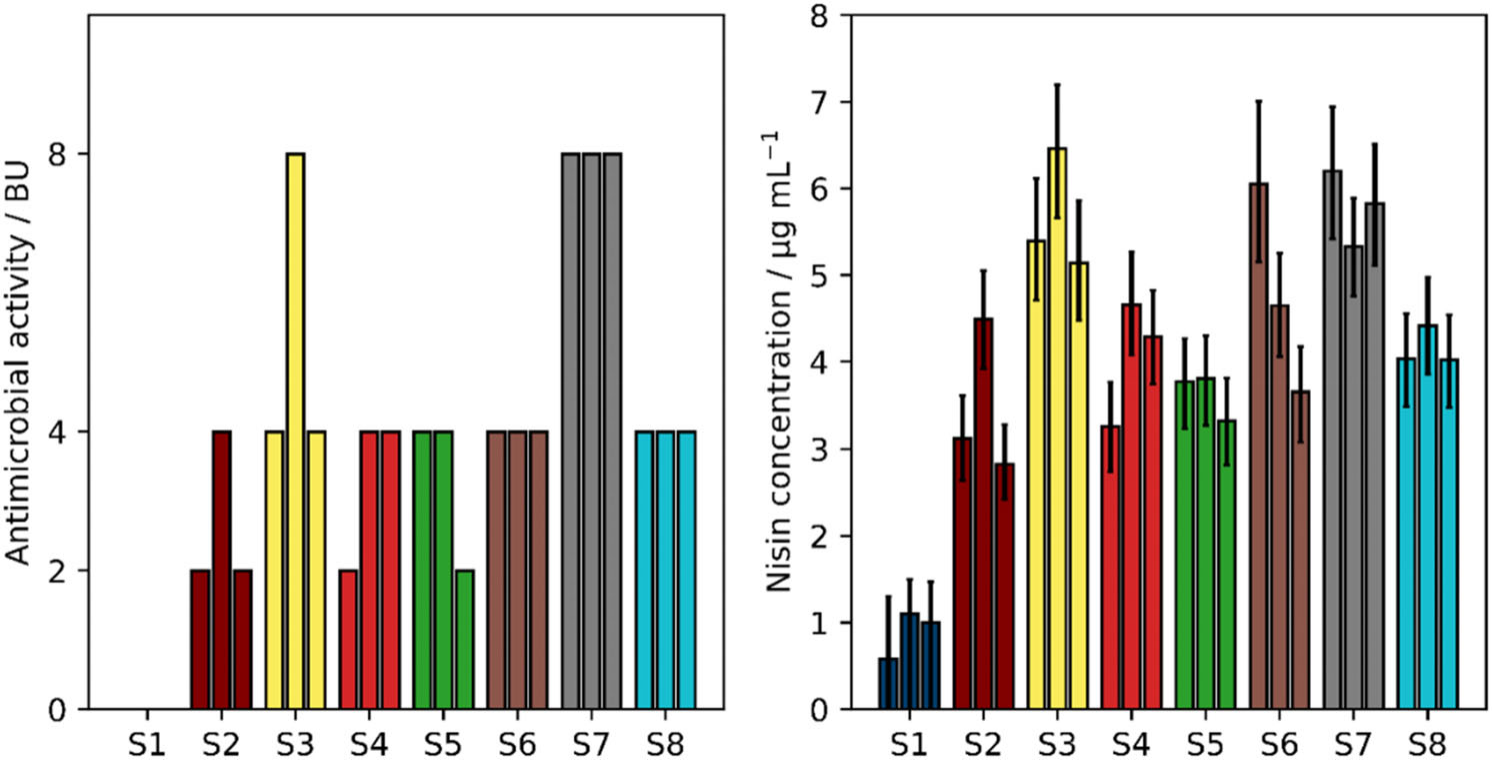
Comparison of evaluation methods for pHluorin2 assay readouts for 24 microcultivation samples of *L. lactis* B1629. Biological triplicates are shown for every condition (S1–S8). Undiluted samples were not considered due to interference of complex media. Left: Deduced antimicrobial activities using threshold method. Right: Inferred nisin concentrations using established non‐linear calibration model (Figure 2). Error bars show 99.9% credible intervals and were not cropped.

Supernatants obtained from microcultivations of *L. lactis* B1629, a natural producer of nisin, in different media compositions were used to compare the quantification using either the standard threshold‐based approach in the simplified representation as BU (Figure 3, left), or the novel calibration model and Bayesian inference (Figure 3, right).

For all samples S1 to S8 biological triplicates were performed. In the threshold‐based approach the samples showed BUs between 0 and 8 (Figure 3, left). For S1, the samples from the cultivation without additional glucose, the antimicrobial activity of the triplicates were below the 50% threshold resulting in BU = 0. For S6, S7, and S8 the triplicates resulted in identical BU values. The overall differences in observed antimicrobial activity between the different samples can be attributed to differences in media composition affecting the growth and nisin production of *L. lactis* B1629 (Table 1).^28^ For S2–S5 the triplicates showed some deviation, stressing one of the major drawbacks of the threshold‐based approaches. While the absolute differences in the fluorescence data were small, even these minor differences between measurements can lead to larger discrepancies on the threshold‐based discrete scale, resulting in larger deviations in bacteriocin units. Due to the combination of serial 2‐fold dilution and discrete scale, differences may thus appear larger than they are. Sample S5 is a perfect example for this effect. While the fluorescence ratio of the third triplicate sample in a four‐fold dilution (0.70) was very close to the other two (0.66, 0.66) showing only minor deviation in range of 3%, it leads to a change from BU = 2 to 4, a 100% increase (50% threshold of 0.67).

The advantages of using a calibration model to infer nisin concentrations become apparent very quickly (Figure 3, right). First, the calibration model provides uncertainty quantification for every single sample including S1, since it takes the spread of measurement readouts, described by the calibration model, into account. The depicted credible intervals show the concentration range, in which the sample lies with a probability of 99.9%. The intervals may be asymmetric for lower concentrations, but do not go below the defined lower limit of 0 μg mL^−1^. Second, calibrations are used to quantify nisin concentration on an absolute, continuous scale, avoiding threshold‐based interpretation based on discrete values. Here, the continuous scale showed substantial advantage in terms of calibration quality.

Moreover, deviations between biological replicates can be more accurately assessed. As can be seen for sample S6, threshold‐based methods led to similar BUs, although the underlying data suggest more variance among the replicates. This becomes evident from the deviation between medians of sample S6 derived from the calibration model. A similar observation holds true for sample S3, showing 100% deviation for the second replicate. The evaluation using the calibration model suggests that the medians of the three biological replicates are within the respective 99.9% credible intervals of the other replicates. In contrast, the slightly higher activity for one replicate of S3 led to an antimicrobial activity of 8 BU in the traditional approach, compared to 4 BU for the other replicates, implying a higher increase than is indeed the case.

## 4 CONCLUSION

To the best of our knowledge, this study marks the first application of a non‐linear calibration model and Bayesian statistics for the quantification of bacteriocins with an antimicrobial activity assay. We demonstrated how the application of such a calibration model allows improved discrimination between samples from different process conditions with regards to bacteriocin formation and elicited antimicrobial activity. Since bacteriocins are susceptible to degradation, aggregation or other instabilities, quantification via antimicrobial activity assays offers the added benefit of measuring only those bacteriocins that are in an active state. Other methods of absolute quantification, such as proteomics, are likely unable to distinguish active and non‐active forms. While other forms of calibration have been used to quantify bacteriocin amounts based on antimicrobial activity measurements, Bayesian calibration provides a more accurate and easily interpretable form of uncertainty quantification.

The novel calibration approach is not limited to specific sensor strains in the pHluorin2 assay, the bacteriocin or the microbial producer strain. With latest advances in peptide synthesis, the availability of commercial bacteriocin standards in sufficient quality for analytical application is feasible. For quantification of other bacteriocins or use of other sensor strains, new calibration models can be established for producer/bacteriocin combinations using a dilution series of bacteriocin standard samples. If bacteriocin standards are available, this calibration approach could be easily transferred to a number of future bacteriocin screenings.

## AUTHOR CONTRIBUTIONS

**Valentin Steier:** Conceptualization; investigation; writing – original draft; visualization; data curation; software; methodology; writing – review and editing; formal analysis. **Michael Osthege:** Conceptualization; methodology; writing – review and editing; software; data curation; formal analysis. **Laura M. Helleckes:** Conceptualization; writing – review and editing; software; methodology; formal analysis; data curation. **Maximilian Siska:** Conceptualization; methodology; writing – review and editing; software; formal analysis; data curation. **Eric von Lieres:** Writing – review and editing; supervision. **Wolfgang Wiechert:** Writing – review and editing; resources. **Sebastian J. Reich:** Conceptualization; writing – review and editing. **Christian U. Riedel:** Conceptualization; writing – review and editing; funding acquisition. **Marco Oldiges:** Conceptualization; writing – review and editing; project administration; supervision; resources; funding acquisition.

## CONFLICT OF INTEREST STATEMENT

The authors declare no conflicts of interest.

### PEER REVIEW

The peer review history for this article is available at https://www.webofscience.com/api/gateway/wos/peer‐review/10.1002/btpr.3495.

## Data Availability

The data that support the findings of this study are openly available in GitHub Repository at https://github.com/JuBiotech/Supplement-to-Steier-et-al.-2024.
